# Enhanced levels of asymmetric dimethylarginine in a serum of middle age patients with myelodysplastic syndrome

**DOI:** 10.1186/1756-8722-6-58

**Published:** 2013-08-19

**Authors:** Jana Štikarová, Jiří Suttnar, Kristýna Pimková, Leona Chrastinová-Mášová, Jaroslav Čermák, Jan E Dyr

**Affiliations:** 1 Institute of Hematology and Blood Transfusion, U Nemocnice 1, 128 20, Prague, Czech Republic

## Abstract

Myelodysplastic syndromes (MDS) are hematological malignancies of unclear etiology where oxidative stress may contribute to the pathogenesis. Methylarginines, naturally occurring inhibitors of NO synthase, can increase superoxide generation from uncoupled NO synthase. We found significant increase in concentrations of asymmetric dimethylarginine (0.84 ± 0.32 μmol/L, p = 0.0022) and malondialdehyde (0.77 ± 0.11 μmol/L, p < 0.001) in sera of MDS patients vs controls (asymmetric dimethylarginine: 0.56 ± 0.16 μmol/L, malondialdehyde: 0.52 ± 0.07 μmol/L). On the contrary, nitrites concentrations were significantly decreased in MDS patients (1.71 ± 0.46 μmol/L, p = 0.0028) vs controls (2.16 ± 0.38 μmol/L). We suppose that the oxidative stress in MDS is enhanced due to methylated arginines influence on NO synthase activity impairment.

## To the editor

Myelodysplastic syndromes (MDS) are a heterogeneous group of clonal hematological disorders, characterized by ineffective hematopoiesis and a high risk of transformation into acute myeloid leukemia. It has been determined that oxidative stress plays a role in the initialization and disease progression [1]. The reactive oxygen species may oxidize tetrahydrobiopterin resulting into nitric oxide synthase (NOS) uncoupling and preferential formation to superoxide anion radical. It was found that methylarginines (asymmetric dimethylarginine - ADMA, N^G^-monomethyl-L-arginine - MMA and symmetric dimethylarginine - SDMA), naturally occurring inhibitors of NOS [2], can profoundly increase superoxide generation from uncoupled NOS [3]. Free methylated arginines, capable of inhibiting NOS [4], are formed exclusively by the sequence of methylation of arginine residues of proteins, followed by proteolysis of these proteins. Protein arginine methylation is in mammalian cells carried out by protein arginine methyltransferases (PRMTs); many of them show links to cancer [5].

The subjects of the study are characterized in Table 1. Ethics approval (Ethics Committee of the Institute of Hematology and Blood Transfusion) and informed consent from all subjects were obtained. Serum concentration of oxidative stress marker malondialdehyde (MDA) was estimated using liquid chromatography (Shimadzu, Tokyo, Japan) of its thiobarbituric acid derivative [6]. Methylated arginines were analyzed using HILIC chromatography with MS/MS detection (ABSciex, Framingham, USA) [7]. Nitrites were assayed by chromatography using the fluorescent reaction product with 2,3-diaminonaphthalene [8].

**Table 1:** Baseline characteristics of MDS patients and healthy controls

	**MDS**	**Controls**
^a^Age (years)	43.7 (33–59)	44.3 (37–67)
Male/Female	11/9	8/8
^a^Serum iron [μmol/L]	22.3 (11.4-43.3)	^b^8.5-28
^a^Serum ferritin [μg/L]	641.2 (8.9-1907.3)	^b^15-150

^a^The data are depicted as averages with ranges. ^b^Reference interval.

The concentrations of methylated arginine derivatives, malondialdehyde and nitrites are summarized in the Table 2. We found significantly increased serum concentrations of ADMA, SDMA, MMA, and MDA in sera of MDS patients as compared with healthy donors. The nitrites concentrations were significantly decreased in sera of MDS patients as compared with controls. The concentration of ADMA strongly positively correlated with concentration of MMA (r = 0.87, p < 0.001) and SDMA (r = 0.70, p < 0.001). ADMA concentration moderately positively correlated with MDA concentration (r = 0.50, p = 0.006).

**Table 2:** Concentrations of methylated arginines in sera of MDS patients and healthy controls

	**MDS**	**Controls**	^**a**^**p**
ADMA [μmol/L]	0.84 ± 0.32	0.56 ± 0.16	0.0022**
SDMA [μmol/L]	0.54 ± 0.18	0.42 ± 0.14	0.0361*
MMA [μmol/L]	0.14 ± 0.05	0.10 ± 0.03	0.033*
Homoarginine [μmol/L]	1.77 ± 1.06	2.32 ± 1.26	0.1777
Citrulline [μmol/L]	46.68 ± 14.96	42.19 ± 12.31	0.3298
MDA [μmol/L]	0.77 ± 0.11	0.52 ± 0.07	<0.001***
Nitrites [μmol/L]	1.71 ± 0.46	2.16 ± 0.38	0.0028**

The data are represented as averages ± SD. ADMA, N^G^, N^G^-dimethyl-L-arginine; MMA, N^G^-monomethyl-L-arginine; SDMA, N^G^,N^G’^-dimethyl-L-arginine; MDA, malondialdehyde. ^a^Two-tailed *t*-test was used to compare measured concentrations of analytes in MDS patients with healthy donors. Statistical significance coding: * p < 0.05, ** p < 0.01 and *** p < 0.001.

Our results showed significantly increased oxidative stress even in MDS patients characterized by moderately enhanced iron and serum transferrin concentrations. Resulting shift of overexpressed [9] NO synthase activity in favour of superoxide production at the expense of nitric oxide synthesis (reflected by nitrites concentrations [10]) was further augmented at the presence of methylated arginines. Therefore, oxidative stress in MDS patients could be explained by a positive feedback of both superoxide and methylated arginines on original NOS activity impairment. Moreover, recently proposed PRMT-specific inhibitors [11] might have a therapeutic effect on leukemia also by oxidative stress reduction.

## Abbreviations

MDS: Myelodysplastic syndromes; NOS: Nitric oxide synthase; ADMA: N^G^,N^G^-dimethyl-L-arginine; MMA: N^G^-monomethyl-L-arginine; SDMA: N^G^,N^G’^-dimethyl-L-arginine; MDA: Malondialdehyde; PRMT: protein arginine methyltransferase.

## Competing interest

The authors indicated no potential conflicts of interest.

## Authors’ contributions

JŠ performed LC-MS/MS analysis of methylated derivatives of arginine. JS participated on LC-MS/MS analysis of methylated derivatives of arginine, carried out data analysis and interpretation and wrote the manuscript. KP and LCM performed malondialdehyde and nitrite analysis. JČ provided clinical data and patient samples. JED conceived of the study and wrote the manuscript. Final approval of the manuscript: All the co-authors. All authors read and approved the final manuscript.
